# Molecular identification and biocontrol of ochratoxigenic fungi and ochratoxin A in animal feed marketed in the state of Qatar

**DOI:** 10.1016/j.heliyon.2023.e12835

**Published:** 2023-01-06

**Authors:** Fatma Ali Alsalabi, Zahoor Ul Hassan, Roda F. Al-Thani, Samir Jaoua

**Affiliations:** Environmental Science Program, Department of Biological and Environmental Sciences, College of Arts and Sciences, Qatar University, 2713, Doha, Qatar

**Keywords:** Ochratoxin A, Animal feed, Toxigenic fungi, Molecular identification, Biocontrol, Qatar

## Abstract

Ochratoxin A (OTA) is a toxic fungal metabolite produced by some *Aspergillus* and *Penicillium* species. This work was designed to explore the presence of OTA and ochratoxigenic fungi in feed grains marketed in Qatar and their biological control by a bacterium (*Burkholderia cepacia*). Significantly higher levels of OTA were detected in mixed grains samples (144.59 ± 6.63 μg/kg), compared to the maize (25.27 ± 1.89 μg/kg) and wheat (3.37 ± 0.11 μg/kg). OTA-producing fungi (*A. niger*, *A. ochraceus*, *A. westerdijkiae*, *A. carbonarius* and *P. verrucosum*) were identified on the basis of their morphological features as well as through polymerase chain reaction (PCR). Putative ochratoxigenic polyketide genes in these isolates were evidenced by using primers AoOTA-L/AoOTA-R (in *A. ochraceus* and *A. westerdijkiae*), AoPks1/AoPks2 (in *A. niger* and *A. ochraceus*) and PenPks1/Penpks2 (in *P. verrucosum*). On synthetic media, *A. westerdijkiae* showed the highest OTA synthesis (5913 ± 576 μg/kg) than the closely related *A. ochraceus* (3520 ± 303 μg/kg), *A. carbonarius* (3064 ± 289 μg/kg) and *P. verrucosum* (3030 ± 710 μg/kg). *Burkholderia cepacia* cells and culture extract showed promising biological control potentials against OTA producing fungi. On the basis of these findings, it can be concluded that animal feed samples are generally contaminated with OTA-producing fungi as well as OTA, and *Burkholderia cepacia* CS5 exhibits promising antifungal activities.

## Graphical abstract

**Figure:**
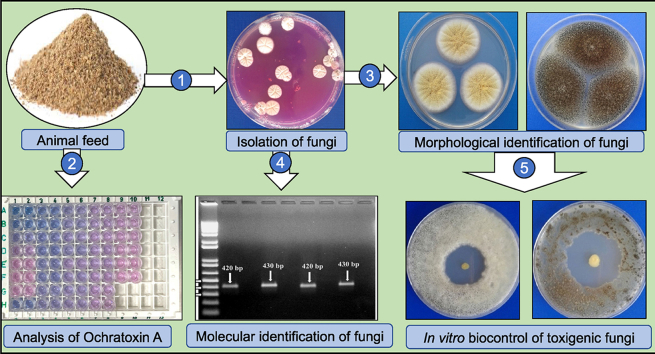

## 1 Introduction

Mycotoxins are secondary fungal metabolites and important contaminants of food and feed throughout the world [1]. The ubiquitous nature of toxigenic fungi has made mycotoxins as unavoidable contaminants of agricultural products and food chains [[2], [3], [4]]. In the list of more than 400 mycotoxins; aflatoxins, ochratoxins, zearalenone, fumonisin and deoxynivalenol (DON) are well known for their toxicities [5,6]. Human exposure to mycotoxins results directly from consumption of mycotoxins contaminated cereals and/or ingestion of animal products such as meat, milk and eggs contaminated with mycotoxins [[7], [8], [9], [10]].

Ochratoxin A (OTA) is mainly produced by toxigenic *Aspergillus* and *Penicillium* spp. including *A. westerdijkiae*, *A. ochraceus*, *A. niger*, *A. carbonarius* and *A. steynii*, *P. verrucosum* and *P. viridicatum* [11]. OTA is primarily a nephrotoxic agent, with a range of toxicities including teratogenic, immunosuppressive, growth retardation and possible carcinogen to humans [12,13]. To safeguard the human population from the risks associated with OTA consumption, the European Union (EU) has set a maximum permissible limit of 250 μg/kg in feed cereals [14]. The confirmed OTA biosynthesis pathway starts with the polyketide synthase (PKS) involving enzymes and intermediate products. At the beginning of the process, acetyl coenzyme A (acetyl-CoA) and malonyl-CoA are converted by a polyketide synthase (OtaA) to 7-methylmellein. This intermediate product (7-methylmellein) is converted by OtaC (a cytochrome P450 monooxygenase) to its oxidized form OTβ. At this step, OtaB (a nonribosomal peptide synthetase) combines OTβ with l-β-phenylalanine to produce OTB. The chlorination of OTB by OtaD (a halogenase) leads to the final product OTA [15,16].

Mycotoxigenic fungi are conventionally identified on the basis of their colony morphology, such as their size, shape, color, sporulation, etc., as well as microscopic examination of fungal hyphae and spores [11]. These morphological identification techniques are not only laborious, but also less precise in terms of morphologically similar fungi. On the other hand, molecular identification of toxigenic genes is gaining popularity because it is less time consuming and most efficient [17]. Specie-specific PCR primers are being designed for precise identification of fungi even if these are similar in morphological characteristics [18].

Prevention of mycotoxins contamination in animal feed at pre-harvest stages is possible by adapting good agricultural practices, using fungal resistance crop varieties and regularly applying fungicides [19]. At post-harvest stage, proper temperature, humidity and water activity are required to avoid the fungal spore germination and thus mycotoxin production [20]. To counter the incidence of mycotoxins and myotoxicities, strategies including chemical (fungicides, pesticides, H_2_O_2_, Ozone gas), physical (irradiation, binding agents like clays, charcoal), and biological (yeast, bacteria, and fungi) have been tested with variable success [[21], [22], [23]].

Biological control of OTA-producing fungi and OTA by using bacterial strains during *in vitro* and *in vivo* experiments has shown promising activities. In a study, Gram-negative soil bacterium *Phenylobacterium immobile* showed the ability to degrade 0.1 mg OTA per liter of culture medium within 5 h into a less toxic carboxylic acid derivative [24]. Likewise, *Bacillus* and *Lactobacillus* species showed their ability to degrade 94% of OTA in culture media [25]. *Bacillus subtilis* is also known to possess powerful antifungal activity by producing antifungal compounds including chitosanase [26]. In our recent studies, *B. licheniformis* [27], *B. pumilus* [28] and *B. megaterium* [29] significantly inhibited the growth of mycotoxins producing fungi as well as their mycotoxin synthesis potential by releasing antifungal molecules. The application of microbes and/or their antifungal molecules in the food and agriculture industry by replacing the synthetic fungicides is definitely of huge significance for human health as well as environmental safety. However, there are certain questions (associated with application of microbes in food) that need answers, such as, i) are the biocontrol microbes equally efficient as the existing synthetic compounds? ii) are the food products safer, nutritious and their sensory characteristics are preserved after application of microbe/microbial molecules? and c) are there any chances of developing resistance in fungal species exposed to biocontrol microbes? Keeping in view all these concerns, only few bacterial and yeast strains have been approved for their direct application of food for their antifungal activities [30].

The present study aimed to investigate the presence of OTA-producing fungal strains and OTA in the animal feed samples collected from the markets in Qatar. Moreover, morphological and molecular approaches were used to identify the OTA-producing fungal strains. The mycotoxins production potential of the fungal strains was explored by investigating OTA-biosynthesis pathways genes as well as *in vitro* OTA production on synthetic media. Finally, the *Burkholderia cepacia* strain CS5 was applied to investigate its potential to inhibit the growth of OTA-producing fungi.

## 2 Materials and methods

### 2.1 Samples collection

A total of 35 animal feed samples including maize (n = 12), wheat (n = 10) and mixed grains (n = 13) were collected randomly from a feed market located in Doha-Qatar. All the samples were collected during the months of August to January. The selection of samples was based on the local livestock farmers preference data gathered from the animal feed dealers. Feed grains included in this study were imported from the different countries including South-Eastern, South African and Middle Eastern countries. In all cases, the constituting grains of mixed-grains feed samples belonged to different countries with diverse geographical and climatic conditions. After grinding, each sample was preserved in airtight bags at 4 °C for the determination of ochratoxin A contents and ochratoxigenic mycobiota. *Burkholderia cepacia* strain CS5 used in this study is our previous soil isolate [31] from the Centre of Biotechnology of Sfax (Tunisia).

### 2.2 Exploration of ochratoxin A contamination

OTA contents of animal feed samples were determined by enzyme linked immunosorbent assay (ELISA) kits [18] obtained from RidaScreen® Ochratoxin A 30/15, Art. No. R1312, R-Biopharm-Germany. Briefly, after grinding all the feed samples, 2 g of each sample were weighted in centrifuge tubes. Following the manufacturer instructions, 5 mL of 1 N HCl was added in each tube and was placed in a shaking incubator for 5 min. In total, 10 mL of Dichloromethane (DCM) was added in each tube, and after centrifugation, lower DCM layer was filtered and equal volume of 0.13 M NaH_2_CO_3_ was added before loading onto ELISA wells. Multiskan FC® microplate reader (Thermo Scientific) was used to obtain the absorbance at 450 nm. For the calculation of OTA levels in unknown samples, a standard curve was generated on the basis of absorbances of six standard OTA solutions provided with the kits. RIDA → SOFT Win software of R-Biopharm-Germany was used for this purpose.

### 2.3 Isolation and identification of ochratoxigenic fungi in animal feed sample

#### 2.3.1 Morphological identification of OTA-producing *Aspergillus* and *Penicillium* spp.

Isolation, enumeration and morphological identification of fungi was performed using established techniques in our lab [18]. Briefly, feed samples were diluted 1:9 in sterile distilled water and 100 μL of samples were spread inoculated on three dichloran – rose bengal – chloramphenicol (DRBC) agar plates. After 4 day of incubation at 28 °C, isolated colonies were counted, and colony forming units (CFU)/g of each feed sample was calculated. With the help of sterile needle picks, fungal colonies were transferred to potato dextrose agar (PDA) plates and incubated for 5 days. Fungal spore suspensions were made in soft agar (0.2% agar in distilled water) containing Tween 80 (0.05%) for declustering of fungal spores. Fungal spores were inoculated on three points [11] on the identification media (Czapek yeast extract agar (CYA), 25% glycerol nitrate agar (G25 N) and malt extract agar (MEA). Colony morphology of fungi were observed after 7 days of incubation and characteristics like color (observe and reverse), shape, sporulation and diameters were recorded and compared with identification key [11]. Relative density [18] of ochratoxigenic fungi was calculated by the formula given below in Eq. (1).

(1) $$RD\;\left(\%\right)=\frac{No.\;of\;isolates\;of\;ochratoxigenic\;specie}{Total\;No.\;of\;fungi\;isolated}\times 100$$

#### 2.3.2 Molecular identification of ochratoxigenic fungi

Selected ochratoxigenic fungi identified on the morphological basis were subjected to molecular identification. Fungal DNA was extracted by using DNeasy® Plant Mini Kit - QIAGEN. For this purpose, fresh fungal colonies on PDA were scraped with scapula and ground to powder with liquid Nitrogen. Following the manufacturer instructions, DNA was extracted and precipitated in ethanol. ITS region was amplified using ITS1-ITS4 primers for checking the suitability of extracted DNA for PCR reactions [32].

Extracted DNA samples were used in PCR reaction using species-specific PCR primers listed in Table 1. PCR reactions were performed in tubes with total reaction volume of 25 μL using Applied Biosystems 96 wells Veriti Thermal Cycler (Thermo Fisher Scientific.

**Table 1:** List of primers used is this study for the molecular identification of fungi and their *pks* genes.

Name	Sequence	Target	Amplicon size (bp)	Reference
**ITS1**	TCC GTA GGT GAA CCT GCG G	Universal fungal primer	550	[32]
**ITS4**	TCC TCC GCT TAT TGA TAT GC			
**OCRAF**	CTTTTTCTTTTAGGGGGCACAG	*A. ochraceus*	430	[17]
**OCRAR**	CAACCTGGAAAAATAGTTGGTTG			
**CAR1**	GCATCTCTGCCCCTCGG	*A. carbonarius*	420	[33]
**CAR2**	GGTTGGAGTTGTCGGCAG			
**WESTF**	CTTCCTTAGGGGTGGCACAG	*A. westerdijkiae*	430	[17]
**WESTR**	CAACCTGATGAAATAGATTGGTTG			
**ITS1**	TCCGTAGGTGAACCTGCGG	*A. niger*	420	[34]
**NIG**	CCGGAGAGAGGGGACGGC			
**AoOTA-L**	CATCCTGCCGCAACGCTCTATCTTTC	*pks* gene	490	[35]
**AoOTA-R**	CAATCACCCGAGGTCCAAGAGCCTCG			
**AoPKS1**	CAG ACC ATC GAC ACT GCA TGC	*pks* gene	549	[36]
**AoPKS2**	CTG GCG TTC CAG TAC CAT GAG			
**Penpks1**	GTC TTC GCT GGG TGC TTC C	*pks* gene	395	[36]
**Penpks**	AGC ACT TTT TCC CTC CAT CTA			

Waltham, MA USA). PCR reaction mixes were prepared by adding genomic DNA, Taq reaction buffer, MgCl2, dNTPs, forward and reverse primers and water [18]. PCR reaction conditions for *A. ochraceus*, *A. westerdijkiae*, *A. carbonarius*, *A. niger* and *A. carbonarius* were set as described previously [17,33,34].

### 2.4 Determination of the ochratoxigenic production potential of fungi

#### 2.4.1 Identification of putative polyketide synthase (*pks*) genes

Based on the morphological and molecular identification, fungal DNA samples were further tested for the putative ochratoxigenic polyketide synthase (*pks*) genes. For this purpose, ochratoxigenic potential of *A. ochraceus* and *A. westerdijkiae* was confirmed by AoOTAL/AoOTAR primers [35]. Likewise, the ochratoxigenic potential of *A. niger* and *A. ochraceus* was confirmed by AoPKS1/AoPKS2 primers [36]. On the other hand, *P. verrucosum* was confirmed by penpks1/penpks2 primers [36] for having ochratoxigenic potential. The *pks* genes were amplified through AoOTA-L/AoOTA-R [35] and AoPKS1/AoPKS2 [36] primer pairs.

#### 2.4.2 I*n vitro* production of OTA on synthetic media

In order to further confirm the OTA-production potential of fungi, agar plug method was adapted [37]. Briefly, fungal strains were inoculated on Yeast Extract Sucrose Agar (YES) media for 7 days at 28 °C. Three colonized media plugs were removed and extracted for OTA contents and analyzed by ELISA assay [38].

### 2.5 Biological control activities of *Burkholderia cepacia* strain CS5

#### 2.5.1 Antifungal activities of *Burkholderia cepacia* in co-incubation assay

In these experiments, *Burkholderia cepacia* CS5 was used to explore its antifungal activities against ochratoxigenic fungi. In the spore overly method, bacterial strain was inoculated at the middle of LB media plates with the help of sterile needle, and incubated for 48 h at 30 °C [38]. Fungal spores at the final concentration of 10^6^/mL were suspended in molted PDA (3 mL). The spores were assayed around the bacterial colonies and incubated at 28 °C for 3 days. The diameter of the fungal zone of inhibition around the bacterial colonies was measured in mm. These assays were performed thrice against all five (*A. ochraceus*, *A. westerdijkiae*, *A. carbonarius*, *A. niger*, *Penicillium verrucosum*) ochratoxigenic species isolated from the animal feed samples.

#### 2.5.2 Antifungal activities of *Burkholderia cepacia* CS5 culture extract

To obtain culture extract of *Burkholderia cepacia* CS5 strain, from a preculture of bacteria, cells were transferred to Nutrient broth in 50 mL tube and incubated for 48 h. After centrifugation, supernatant was separated, and filter sterilized using 0.44 μm syringe filter. Molten PDA was added with the bacterial culture extract at 0%, 0.5%, 1% and 2%. From each ochratoxigenic fungi (*A. ochraceus*, *A. westerdijkiae*, *A. carbonarius*, *A. niger*, *Penicillium verrucosum*), spores were inoculated at the center of the plates and incubated for 5 days. The colony diameter of fungi was measured (in mm) and compared with the control to calculate fungal growth inhibition ratio as below in Eq. (2).

(2) $$Inhibition\;ration\;\left(\%\right)=\frac{diameter\;of\;control-diameter\;of\;treated}{diameter\;of\;control}\times 100$$

### 2.6 Statistical analysis

Data was analyzed using one-way analysis of variance (ANOVA) test. Means in different groups were compared using *Post hoc* multiple comparison with Fisher's least significant difference (LSD) test. All analyses were performed using the statistical package for social sciences (SPSS) Version 23.0 for this purpose.

## 3 Results and discussion

### 3.1 Levels of mycotoxins in animal feed

Ochratoxin A (OTA) is one of the most extensively researched mycotoxins due to its toxic effects on animals and humans [39,40]. In the present study, levels of OTA were significantly higher (*P* < 0.01) in the mixed-grains samples compared to maize and wheat samples. The levels of OTA in mixed grains, maize and wheat samples were 144.59 ± 6.63, 25.27 ± 1.89 and 3.37 ± 0.11 μg/kg, respectively. In all the tested samples, levels of OTA were lower than the maximum permissible limit of 250 μg/kg set by the European Commission [14]. The presence of OTA in cereal is generally associated with their grain quality and physical condition [18,39]. In Italy, all the tested poultry feed samples contained a varying range of OTA from 0.04 to 6.50 μg/kg [41]. Similarly, in a study in Brazil [42], about 31% of swine feed samples were contaminated with OTA with levels ranging from 36 to 120 μg/kg. In unmixed cereals and grains, the mean concentration of OTA was at the range of 1–100 μg/kg [43]. Although the levels of OTA detected in the animal feed samples were within the safer limit, suggesting that the feed grains marketed in Qatar are of acceptable quality without anticipated effects on animal health and productivity. However, the further studies on the simultaneous occurrence of multi-mycotoxins in feed could help investigate if any synergistic toxicities are expected.

### 3.2 Isolation and identification of toxigenic fungi

#### 3.2.1 Morphological identification ochratoxigenic fungi

**Fig. 1:**
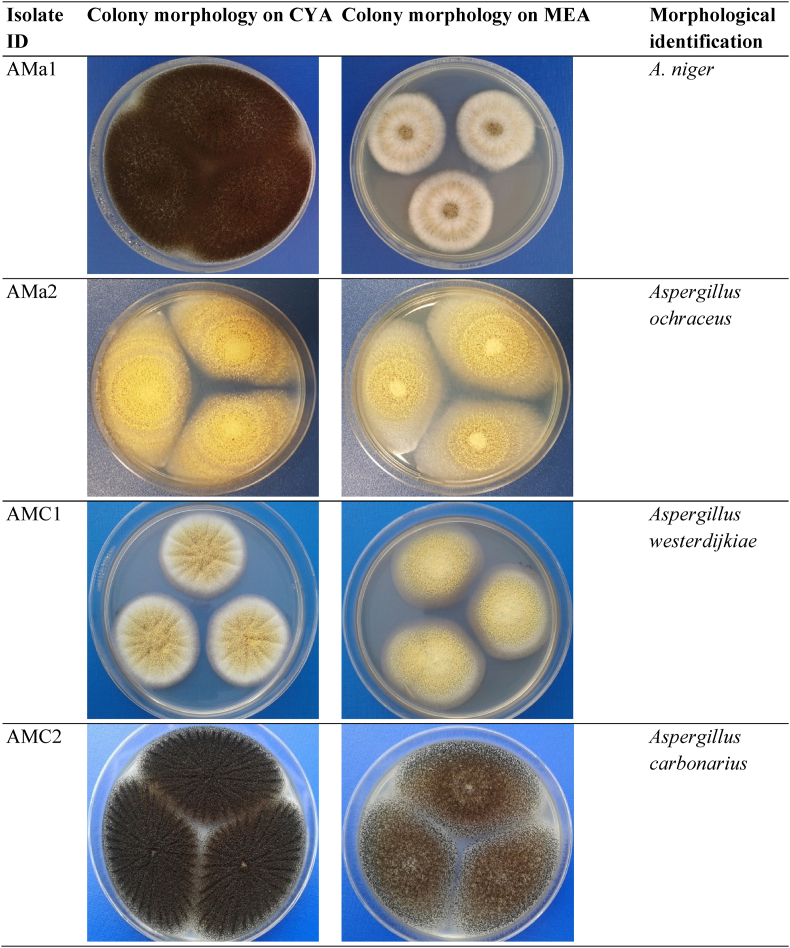
Colony morphology of ochratoxigenic fungi isolated from the animal feed samples.

The presence of OTA in the animal feed observed in Section 3.1, suggested the occurrence of ochratoxigenic fungi. The fungal CFU/g determined on DRBC showed highest in mixed cereals (2910 ± 121) followed by maize (1943 ± 129) and wheat (1121 ± 90) samples. In line with the present findings, in a previous study [18] there was a significantly higher fungal contamination (3210 ± 89 CFU/g) in mixed cereal feed as compared to maize (2445 ± 110 CFU/g) and wheat grains (2013 ± 57 CFU/g). Based on the morphological characteristics on CYA and MEA, ochratoxigenic [11] *Aspergillus* and *Penicillium* spp., were detected in all three types of feed samples (Fig. 1). The relative density (RD) mycotoxigenic fungi was 9–50% with highest being for *A. niger* (50%), *A. ochraceus* (12%), *A. westerdijkiae* (9%), *A. carbonarius* (15%), *P. verrucosum* (14%). The presence of mycotoxigenic fungi in the marketed food and feed in Qatar have been reported in several studies [18,44]. The presence of *Aspergillus* and *Penicillium* as major contaminating fungi in the marketed feed samples is probably associated with the origin of the feed samples. In a country like Qatar, feed cereals are imported mainly from the countries of tropical climate regions, where the occurrence of toxigenic *Aspergilli* and *Penicillium* is commonly reported due to favorable climatic conditions [45].

#### 3.2.2 Molecular identification of ochratoxigenic isolates

**Fig. 2:**
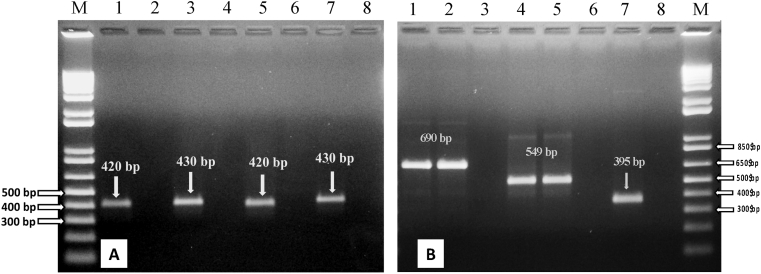
Molecular identification of ochratoxigenic fungi and exploration of OTA cluster genes (Supplementary file Fig. 2). (A) PCR amplification of fungal DNA using species-specific primers. Lanes; 1, primer ITS1/NIG with DNA from *A. niger* (amplicon size 420 bp); 3, primer OCRAF/OCRAR with DNA from A. ochraceus (amplicon size 430 bp); 5, primer CAR1/CAR2 with DNA from A. carbonarius (amplicon size 420 bp); 7, primer WESTF/WESTR with DNA from A. westerdijkiae (amplicon size 430 bp). Lanes; 2, 4, 6 and 8 represent non-template control of their pervious lanes. (B) PCR amplification of ochratoxigenic pks genes in different isolates. Lanes; 1 and 2, DNA from *A. ochraceus* and *A. westerdijkiae* with primer AoOTAL/AoOTAR; 4 and 5, DNA from *A. niger* and *A. ochraceus* with primer AoPKS1/AoPKS2; 7, DNA from *P. verrucosum* with primer penpks1/penpks2. Lanes 4, 7 and 8 indicate non-template control of their previous lanes. Lane M (both in A and B); 1 kb plus DNA marker with fragment sizes 12,000, 11,000, 10,000, 9000, 8000, 7000, 6000, 5000, 4000, 3000, 2000, 1000, 850, 650, 500, 400, 300, 200, 100 bp.

The confirmation of selected ochratoxigenic isolates was performed through PCR analysis using specie-specific primers (Fig. 2A). *A. niger* was identified using ITS1/NIG primers [34] giving a single PCR DNA fragment of 420 bp. The morphological identification for these isolates matched with their PCR based identification. DNA samples of *A. ochraceus* were used in PCR using OCRAF/OCRAR primers, and showed a single PCR DNA fragment of 430 bp [17]*. A. westerdijkiae* was molecularly identified by PCR using WESTF/WESTR primers, with a single amplification fragment size of 420 bp [17]. *A. carbonarius* isolated were identified using CAR1/CAR2, which showed a single PCR DNA fragment of 430 bp [33]. For, *P. verrucosum* the specific species primer was not available, so *pks* gene specific primer for *P. verrucosum* was used for both species molecular identification and the identification of toxin coding genes. A summary of all the PCR outcomes is presented in Table 2.

**Table 2:** Summary of PCR amplification for the molecular identification of fungal isolates and presence of putative ochratoxigenic *pks* gene.

**Isolate code**	ITS1/ITS4	ITS1/NIG	OCRAF/OCRAR	WESTF/WESTR	CAR1/CAR2	AoOTA-L/AoOTA-R	AoPks1/AoPks2	Penpks1/Penpks2
AMa1	+	+	–	–	–	+	–	–
AMa2	+	–	+	–	–	+	+	–
AMC1	+	–	–	+	–	+	–	–
AMC2	+	–	–	–	+	–	–	–
PWT1	+	–	–	–	–	–	–	+

### 3.3 Ochratoxin A synthesis potential of the isolates

#### 3.3.1 Exploration of cluster genes involved in mycotoxin synthesis

Ochratoxin A production potential of the fungal isolates was explored by PCR using polyketide synthase gene (*pks*) specific primers. PCR amplified fragments confirmed the ochratoxigenic fungi and putative ochratoxigenic *pks* genes in the fungal isolates. Many studies confirmed that OTA biosynthesis pathways in *Penicillium* and *Aspergillus* species are regulated by PKS and NRPS genes [46]. Based on the latter, the nature of polyketide synthase gene (*pks*) in *A. niger*, *A. ochraceus*, *A. westerdijkiae* and *P. verrucosum*, confirmed their ochratoxigenic potential (Fig. 2B). The ochratoxigenic potential of *A. ochraceus* and *A. westerdijkiae* was further confirmed using AoOTAL/AoOTAR primers [35], and the DNA fragment of *pks* gene appeared on the gel with size of 690 bp. The ochratoxigenic potential of *A. niger* and *A. ochraceus* was confirmed using AoPKS1/AoPKS2 primers [36] by obtaining a fragment of this *pks* gene of 549 bp. On the other hand, *P. verrucosum* was confirmed, using penpks1/penpks2 primers [36] giving a fragment of 395 bp, for having ochratoxigenic potential. The *pks* gene was found in *A. ochraceus* by using two primer pairs. It was evidenced using AoOTAL/AoOTAR and AoPKS1/AoPKS2 pairs, which suggests the presence of different gene sequences that activate the OTA synthesis in *A. ochraceus*. Using available primers, the toxigenic (*pks*) gene of *A. carbonarius* was not amplified, although *A. carbonarius* has shown toxigenic potential as confirmed by culture plugs method (Section 3.2.2).

#### 3.3.2 Determination of ochratoxin A producing potential of isolates by agar-plug method

**Fig. 3:**
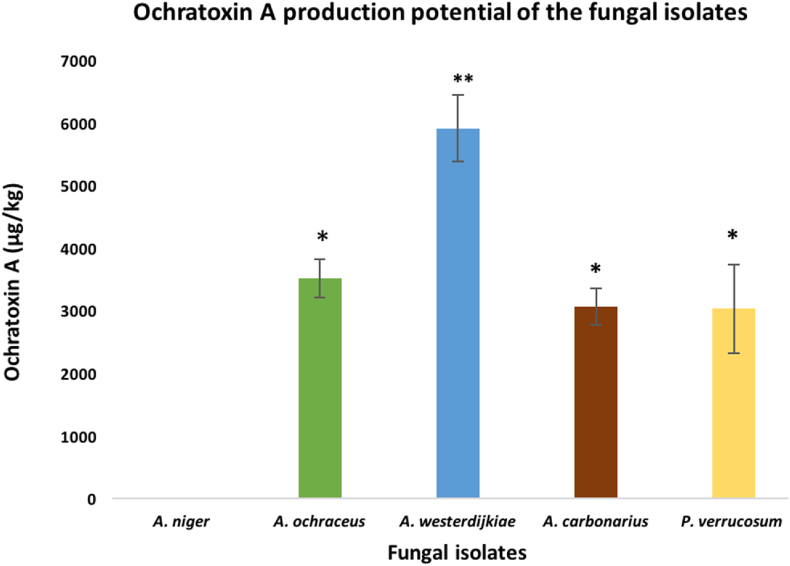
In vitro OTA production potential of the fungal isolates obtained from the animal feed samples. Each bar indicates the average of 3 values (*P < 0.05, **P < 0.01).

After confirmation of the presence of ochratoxigenic *pks* gene in fungal isolates, all fungal isolates (*A. niger*, *A. ochraceus*, *A. westerdijkiae*, *A. carbonarius* and *P. verrucosum)* were tested for their ochratoxigenic potential using culture plugs method [37]. OTA contents of colonized media plugs analyzed by ELISA showed the production of mycotoxins by all tested fungal isolates except *A. niger*. A significantly higher level of OTA (5913 ± 976 μg/kg) was detected in the agar plugs associated with *A. westerdijkiae* (Fig. 3). Several studies have reported *A. westerdijkiae* as a major OTA-producer [18,47]. *A. ochraceus*, *A. carbonarius* and *P. verrucosum* showed a non-significant difference in their OTA production potential. The toxigenic potential of *A. carbonarius* was not confirmed using available primers, but culture plugs method confirmed that *A. carbonarius* has OTA production potential with an average concentration of 3064 ± 213 μg/kg. *A. carbonarius* isolates have been reported in many commodities for their ability to produce OTA, but level of production depends mainly on the temperature conditions, where the cooler temperature, 15–20 °C is the optimum for OTA production; as the temperature goes higher as OTA production become less or inhibited [11]. Interestingly, *in vitro* OTA production potential of the fungal isolates had a positive correlation with the levels of OTA in their host commodity; *A. westerdijkiae* that have the higher OTA production potential, was isolated from mixed cereals animal feed sample, which was contained the highest level of OTA. The levels of OTA synthesis by different *A. ochraceus* isolates were influenced by the temperature and the nature of substrate [48]. The highest production was achieved at 20 °C on green coffee beans [48]. The distribution of *P. verrucosum* is usually restricted to cool areas [11], but its occurrence in the warm environment of Qatar, may reduce its OTA production potential. The absence of OTA synthesis by *A. niger* could be due to lack of certain environmental conditions needed for OTA production. Most of *A. niger* isolates from other studies were not able to produce OTA and only few isolates were able to produce OTA under very specific conditions [11] The levels of OTA accumulated by toxigenic fungi are higher than those observed in Brazil [42], where *A. ochraceus* and *P. verrucosum* isolates produced 25–120 μg/kg of OTA. In the present study, the lowest OTA level of 3030 μg/kg was observed *P. verrucosum*. Ochratoxins are globally distributed secondary metabolites that are produced mostly by some toxigenic groups of *Penicillium* and *Aspergillus* that infect many raw agricultural products [49]. Initially, OTA production was thought to be synthesized only by *P. verrucosum* and *A. ochraceus*. However, recent surveys have demonstrated certain species from the black *Aspergilli*, such as *A. carbonarius* and *A. niger* contribute to OTA production in foodstuffs such as dried vine fruits, wine, and grapes [50,51].

### 3.4 Biological control of ochratoxigenic fungi though *Burkholderia cepacia* strains

#### 3.4.1 Antagonistic activities of *B. cepacia* strain CS5 in co-incubation assay

**Fig. 4:**
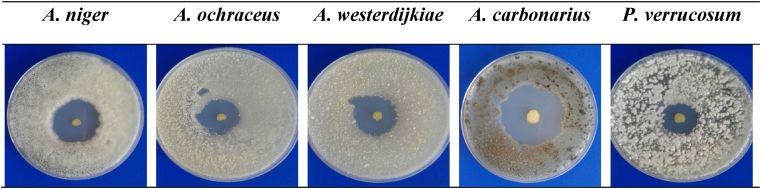
Antifungal activity *Burkholderia cepacia* CS5. Each bar indicates the average of 9 values (*P < 0.05).

All fungal isolates showed sensitivity to diffusible compound(s) of *Burkholderia cepacia* CS5 during co-incubation assay. Among the tested fungi, *A. carbonarius* showed significantly higher sensitivity to *Burkholderia cepacia* CS5 with an average 40.33 mm zone of inhibition, and *P. verrucosum* was the least with an average 21.42 mm zone of inhibition (Fig. 4). These findings suggest that *Burkholderia cepacia* CS5 is effective against *Aspergillus* and *Penicillium* species. In a previous study, *Burkholderia cepacia* demonstrated a remarkable antifungal activity against *Alternaria alternata*, *Aspergillus niger*, *Fusarium culmorum*, *F. graminearum*, *F. oxysporum* and *Rhizoctonia solani* [31]. *Burkholderia cepacia* is known to play an important role against plant pathogenic fungi by producing several volatile and diffusible antifungal compounds, such as pyrrolnitrin, pyoluteorin, phenazine, 2,4-diacetylphloroglucinol, alericidins and cepacin [31,52,53]. The spectrum of activities of each type of these compounds is different and are produced in different environmental conditions.

#### 3.4.2 Antagonistic activities of *Burkholderia cepacia* cell free culture supernatant

**Fig. 5:**
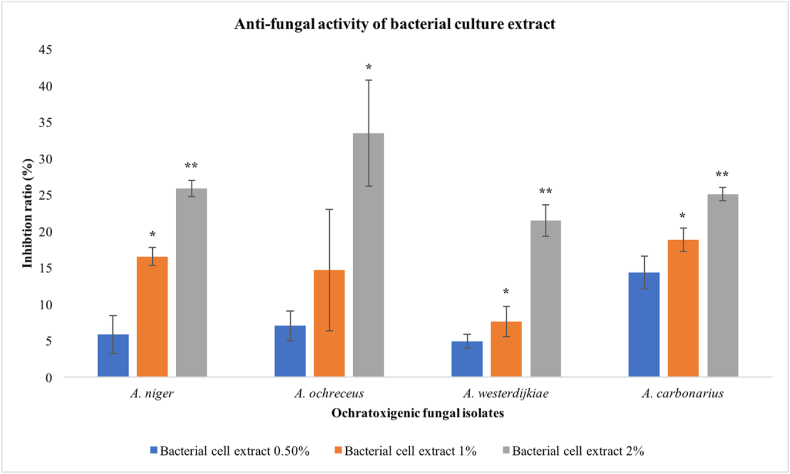
Fungal growth inhibition (%) at different concentrations (0.5%, 1 and 2%) of *Burkholderia cepacia* CS5 culture extract. Each bar indicates the average of 6 values (*P < 0.05, **P < 0.01).

The culture extracts of *Burkholderia cepacia* CS5 were separated and tested in different concentrations (0%, 0.5%, 1% and 2%) against ochratoxigenic fungal isolates. The effect of bacterial extract on the size of fungal colonies (mm) are represented in Table 3 and Fig. 5. *Burkholderia cepacia* CS5 culture extracts had inhibitory effects on all ochratoxigenic fungi except *P. verrucosum*. With increasing the concentration of bacterial culture extract, the fungal colonies dimeter (mm) showed decreasing trend. *A. carbonarius* showed highest sensitivity to CS5 extracts at all concentrations (0.5%, 1% and 2%) followed by *A. niger*, *A. ochraceus* and *A. westerdijkiae*. *A. niger*, *A. westerdijkiae* and *A. carbonarius* showed significantly higher sensitivity (*P* < 0.01) to *Burkholderia cepacia* CS5 bacterial culture extract at 2% than 0.5% and 1%, and *A. ochraceous* showed significantly higher sensitivity (*P* < 0.05) at 2% than 0.5% and 1%. *A. niger*, *A. westerdijkiae* and *A. carbonarius* showed significantly higher sensitivity (*P* < 0.05) to *Burkholderia cepacia* CS5 bacterial culture extract at 1% than 0.5%. *A. ochraceous* showed non-significance differences in the sensitivity at 1% and 5%.

**Table 3:** Effect of *Burkholderia cepacia* CS5 culture extract on the fungal colony size.

Species	Fungal Colony Size (mm) at different concentration of bacterial culture extract (Inhibition %)			
	0%	0.5%	1%	2%
*A. niger*	71.5 ± 0.9	67.3 ± 2.3 (5.8)	59.7 ± 1.0 (16.5)	53 ± 1.1 (25.9)
*A. ochraceus*	54.3 ± 0.8	50.5 ± 0.6 (7.1)	46.3 ± 4.3 (14.7)	36.2 ± 4.2 (33.4)
*A. westerdijkiae*	54.3 ± 0.5	51.7 ± 0.8 (4.9)	50.2 ± 0.7 (7.7)	42.7 ± 1.2 (21.5)
*A. carbonarius*	74.3 ± 0.8	63.7 ± 1.9 (15.3)	60.3 ± 1.0 (18.8)	55.7 ± 0.5 (25.1)
*P. verrucosum*	11.0 ± 0.0	11.0 ± 0.0 (0)	11.0 ± 0.0 (0)	11.0 ± 0.0 (0)

Values are mean ± SD of six independent replicates. Inhibition ratios are calculated with the formula given in the methodology section and values are presented in parenthesis.

The inhibition ratios of *Burkholderia cepacia* CS5 extracts noted in the present study were much lower than those noted with the *Lactobacillus* spp. The cell free culture supernatant of *Lactobacillus plantarum* against *P. expansum* and *A. parasiticus* showed 58% and 73% of the growth inhibition, respectively [54]. This variation in the inhibition ratio could be due to the differences among the fungal species, because some fungi are more resistant to the antifungal products than others [11]. *Burkholderia* genus comprises more than 40 Gram-negative, β-proteobacteria types in different environmental settings/places. They demonstrate that *Burkholderia* is a very effective bioremediation and biocontrol agent by secreting various diffusible and volatile antimicrobial compounds. During the screening experiments, along with several other microbial strains (yeast and bacteria) Burkholderia cepacia was tested for its antifungal activities. Based on the strong antagonistic potential presented by *Burkholderia cepacia* during the initial experiments, this bacterium was selected for further experiments on different ochratoxigenic fungi. However, the precise nature of antifungal molecule(s) produced by this bacterium and their safety profiles in food and feed will guide their application in agriculture industry.

## 4 Conclusion

Feed grains marketed in the state of Qatar are generally contaminated with OTA and OTA-producing fungi. Levels of OTA and fungal burdens were higher in the mixed-grain samples compared with the maize and wheat samples. Although the levels of OTA in all the tested grains were within the EU permissible limit, still there is need for continuous monitoring particularly for the presence of multi-mycotoxins which are known for synergistic toxicities. Molecular tools using specific primers is a quick and less laborious procedure in the identification of toxigenic fungi as well as foreseeing their OTA-synthesis potential. *Burkholderia cepacia* cells and culture extract showed promising biological control potential against OTA-producing fungi, which can be further explored for potential application in field application.
