# Supplementary material for: Molecular identification and biocontrol of ochratoxigenic fungi and ochratoxin A in animal feed marketed in the state of Qatar
Source: Heliyon. 2023 Jan 6;9(1):e12835. doi: 10.1016/j.heliyon.2023.e12835 (PMC9840141; doi:10.1016/j.heliyon.2023.e12835)
Supplement: Multimedia component 1 [file mmc1.docx]

**Supplementary file Figure 2:** Molecular identification of ochratoxigenic fungi and exploration of OTA cluster genes.

**A.** PCR amplification of fungal DNA using species-specific primers. Lanes; 1, primer ITS1/NIG with DNA from *A. niger* (amplicon size 420 bp); 3, primer OCRAF/OCRAR with DNA from *A. ochraceus* (amplicon size 430 bp); 5, primer CAR1/CAR2 with DNA from *A. carbonarius* (amplicon size 420 bp); 7, primer WESTF/WESTR with DNA from *A. westerdijkiae* (amplicon size 430 bp). Lanes; 2, 4, 6 and 8 represent non-template control of their pervious lanes.

**B.** PCR amplification of ochratoxigenic *pks* genes in different isolates. Lanes; 1 and 2, DNA from *A. ochraceus* and *A. westerdijkiae* with primer AoOTAL/AoOTAR; 4 and 5, DNA from *A. niger* and *A. ochraceus* with primer AoPKS1/AoPKS2; 7, DNA from *P. verrucosum* with primer penpks1/penpks2. Lanes 4, 7 and 8 indicate non-template control of their previous lanes. Lane M (both in A and B); 1kb plus DNA marker with fragment sizes 12000, 11000, 10000, 9000, 8000, 7000, 6000, 5000, 4000, 3000, 2000, 1000, 850, 650, 500, 400, 300, 200, 100 bp.
